# Ligand-guided homology modeling drives identification of novel histamine H3 receptor ligands

**DOI:** 10.1371/journal.pone.0218820

**Published:** 2019-06-25

**Authors:** David Schaller, Stefanie Hagenow, Holger Stark, Gerhard Wolber

**Affiliations:** 1 Molecular Design Lab, Pharmaceutical and Medicinal Chemistry, Institute of Pharmacy, Freie Universität Berlin, Berlin, Germany; 2 Institute of Pharmaceutical and Medicinal Chemistry, Department of Pharmacy, Faculty of Mathematics and Natural Sciences, Heinrich-Heine-Universität Düsseldorf, Düsseldorf, Nordrhein-Westfalen, Germany; University of Parma, ITALY

## Abstract

In this study, we report a ligand-guided homology modeling approach allowing the analysis of relevant binding site residue conformations and the identification of two novel histamine H_3_ receptor ligands with binding affinity in the nanomolar range. The newly developed method is based on exploiting an essential charge interaction characteristic for aminergic G-protein coupled receptors for ranking 3D receptor models appropriate for the discovery of novel compounds through virtual screening.

## Introduction

Virtual screening campaigns are typically classified into ligand-based approaches exploiting the similarity of molecules to already known active ligands, and structure-based approaches, where virtual screening models describe three-dimensional chemical interactions between molecules and the target structure [1]. A literature survey revealed that structure-based approaches are on average less successful in identifying highly active hits than ligand-based approaches [2]. However, if active lead compounds are identified, structure-based approaches hold the information for a subsequent rational optimization of interactions between ligand and target structure.

Although the amount of publicly available data for ligand-protein complexes is constantly increasing, structural data is not always available. In this situation researchers often rely on homology modeling, a method for generating the protein structure of interest based on closely related proteins with resolved crystal structures [3]. Including ligand information can aid the homology modeling process and decrease the level of uncertainty by evaluating homology models to enrich known actives from decoys in docking experiments and/or to allow docking poses that match data from mutational studies (often termed ‘ligand-based’, ‘ligand-guided’, ‘ligand-steered’ or ‘ligand-supported homology modeling’). Especially G-protein coupled receptors (GPCRs) were extensively studied using such approaches including serotonin receptors [4], dopamine receptors [5], GABA_B_ receptor [6] and neurokinin receptor 1 [7].

Most of these approaches heavily depend on scoring algorithms employed by docking programs to rank ligand poses and to estimate binding affinity [4–6]. However, docking scores often poorly corelate with binding affinity [8]. Also, searching for or optimizing a single homology model to bind a diverse set of ligands is arguable, since very different ligands might bind to or induce different protein conformations [9]. In contrast, Evers and Klebe avoided the use of docking scores by optimizing a homology model of the neurokinin receptor 1 to allow interactions with a single ligand that was extensively investigated including structure activity relationship of the ligand and mutational studies of the receptor to identify interacting amino acid chains [7]. Though, relying on mutational data can also be misleading, since mutations distant from the protein binding pocket can also drastically affect ligand binding [10].

In this study, we were interested if a single, yet important and reliable interaction can be exploited in a ligand-guided homology modeling workflow for the histamine H_3_ receptor (H_3_R) to gain structural knowledge about the binding site and to guide the selection of a homology model for subsequent virtual screening. We focused on an interaction of charged functional groups between ligands and aminergic GPCRs, which is well characterized and has been observed in multiple crystal structures of different GPCRs [11,12]. H_3_R was selected as target for several reasons: (i) ligand data is publicly available, (ii) crystal structure is currently still missing, (iii) H_3_R is an important drug target discussed for many severe diseases including Alzheimer’s disease, schizophrenia, Parkinson’s disease, narcolepsy, pain, and obesity among others [13,14] and (iv) a recent study of us revealed that H_3_R and melanin-concentrating hormone receptor 1 can be inhibited by the same ligand which could be potentially used in obesity treatment [15]. In this project, 1000 homology models were generated and evaluated for allowing a charged interaction with a defined set of ligands. Best and worst performing models were structurally investigated and revealed the importance of distinct binding site residue conformations for proper ligand docking. The highest ranked model was used for a pharmacophore-based virtual screening campaign and led to the identification of two novel H_3_R ligands with nanomolar affinity.

## Results and discussion

### Ligand-guided homology modeling

**Fig 1:**
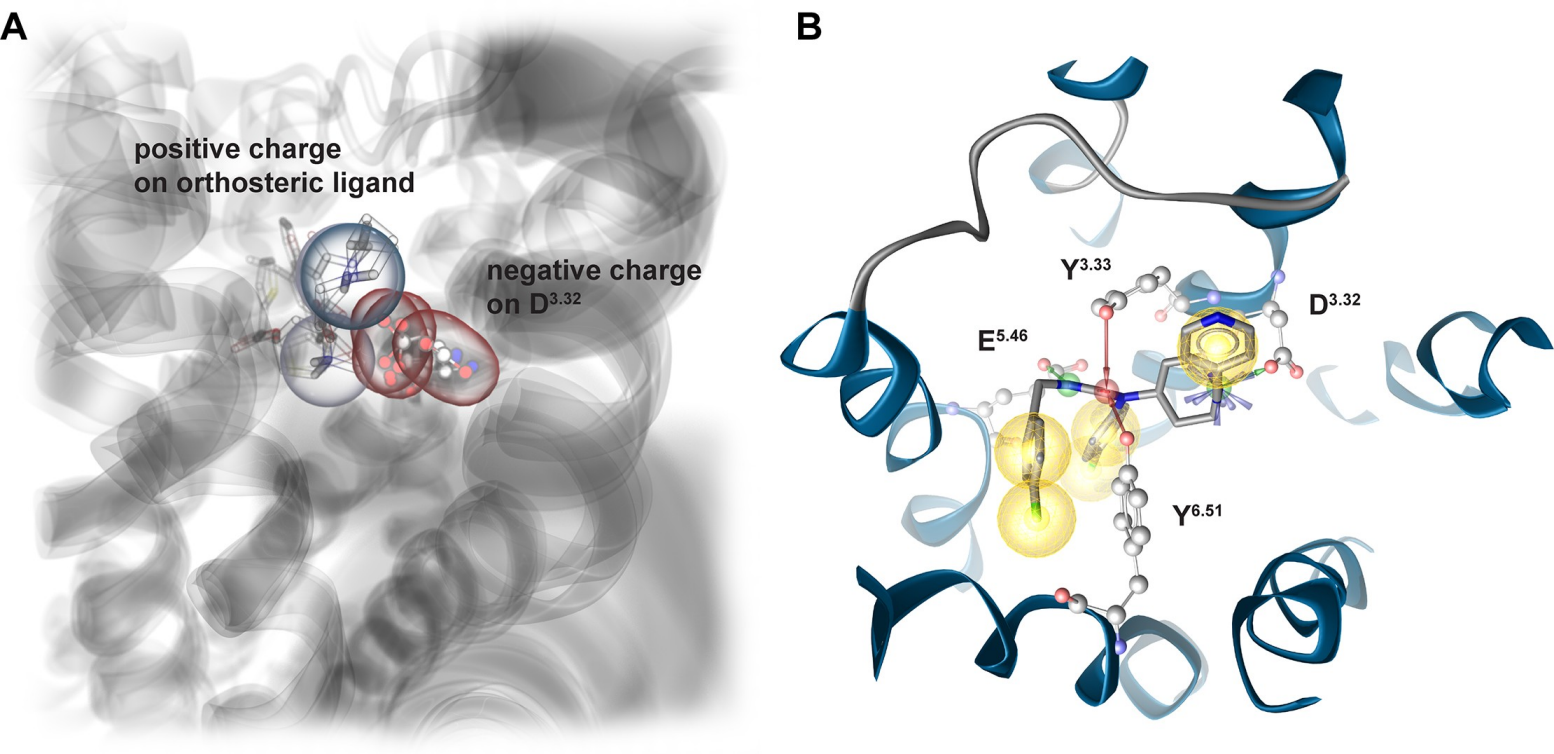
Ligand-guided homology modeling workflow exploits essential charged interaction known from aminergic GPCRs. (A) Aminergic GPCRs show a common charge interaction of highly diverse ligands with Aspartate 3.22 as illustrated for Eticlopride co-crystallized with the dopamine D3 receptor (3PBL [21]), Tiotropium co-crystallized with the muscarinic M_4_ receptor (5DSG [22]) and Carazolol co-crystallized with the β_2_ adrenoceptor (5JQH [23]). (B) Predominant binding mode of ligand series (Table C in S1 File) used for ligand-guided homology modeling. The depicted docking pose of CHEMBL1091834 involves a charged interaction with D_3.32_¸ hydrogen bonds to D_3.32_, Y_3.33_, E_5.46_ and Y_6.51_ as well as several hydrophobic contacts. Red arrows–hydrogen bond acceptors, green arrows–hydrogen bond donors, blue star–positive ionizable, yellow sphere–hydrophobic contact.

A template search revealed that the crystal structure of H_1_R (3RZE [16]) does not show the highest sequence similarity to H_3_R. Also, the extracellular loop 2 close to the orthosteric binding pocket is not resolved in the H_1_R structure. Hence, homology modeling was performed with a multiple-template approach employing crystal structures of H_1_R, muscarinic M_2_ receptor (M_2_R) and muscarinic M_3_ (M_3_R) receptor to generate 1000 homology models of H_3_R with MODELLER 9.15 [17]. The average heavy atom RMSD of 1.2 Å was calculated with VMD 1.9.2 [18], whereat side chain heavy atoms were more flexible (1.6 Å) than backbone heavy atoms (0.4 Å). A set of 9 antagonists [19] (Table C in S1 File) was chosen to guide the selection of a homology model for later pharmacophore studies. We were specifically interested into this ligand series, since we found highly similar molecules active against the melanin-concentrating hormone receptor 1 (MCHR1) and dual antagonism of H_3_R and MCHR1 might present a potential treatment option for obesity [15]. Additionally, these ligands are rather big showing Y-shaped conformations and thus should allow the selection of a homology model with an open binding pocket able to harbor diverse ligands. Subsequently, models were scored for presence of a charged interaction between the docked ligands and D_3.32_ (numbering from Ballesteros-Weinstein numbering scheme [20]), that is known to be essential for ligand binding to aminergic GPCRs (Fig 1A) [11]. Docking and scoring have been performed twice to control for variations introduced by the docking algorithm (Fig B in S1 File). The highest ranked model achieved an average score of 0.835 in both docking experiments. This means that 83.5% of the docking poses allow for a charged interaction with D_3.32_. The predominant binding mode of docked ligands involves a charged interaction with D_3.32_, hydrogen bonds with D_3.32_, Y_3.33_, E_5.46_ and Y_6.51_ as well as several hydrophobic contacts (Fig 1B). Interestingly, we found that 25% of generated models retrieved a score of 0.1 or lower. From these, 7 models had a score of 0, which means that none of the docking poses was involved in the essential charged interaction.

**Fig 2:**
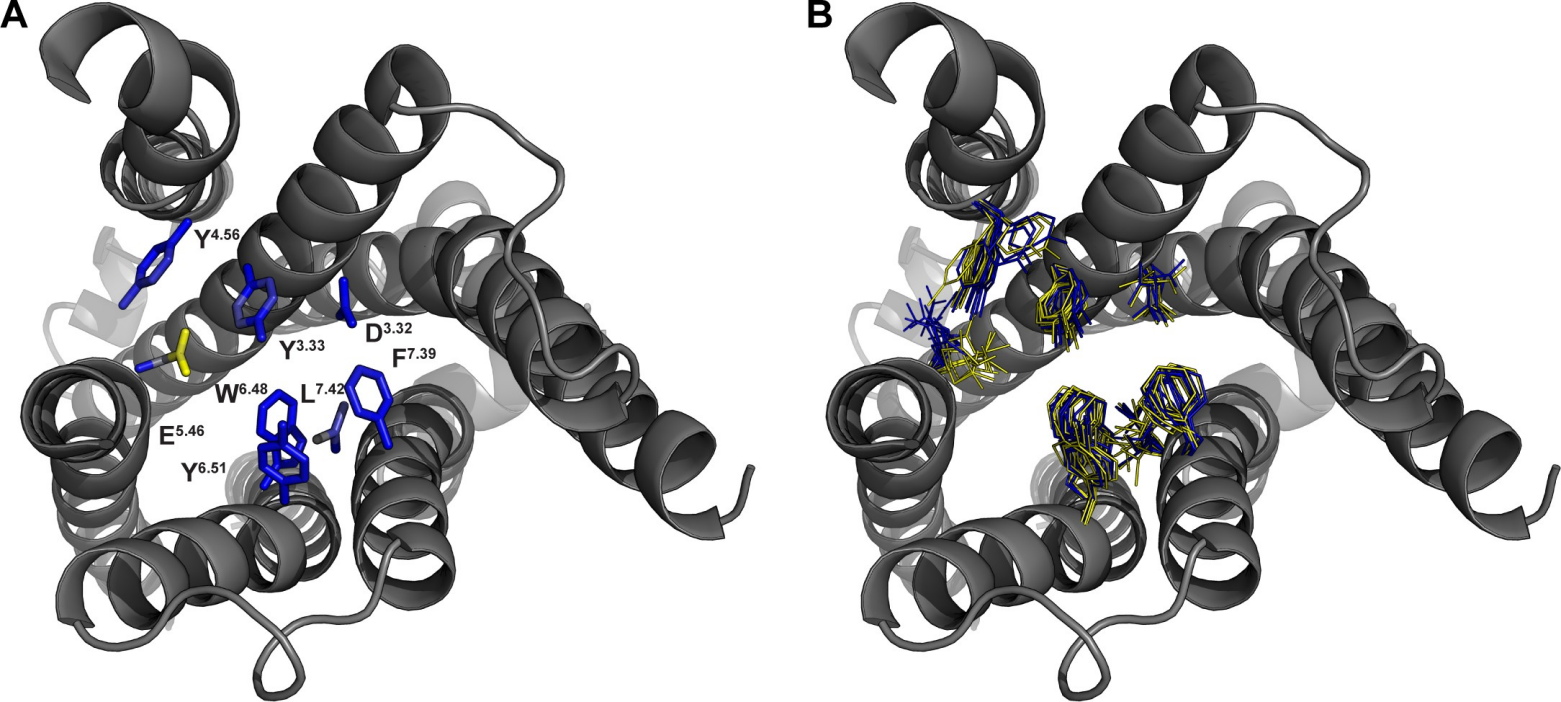
Best and worst scored homology models show distinct structural differences. Top view onto the orthosteric binding pocket of H_3_R. Extracellular loop 2 is not shown for sake of clarity. (A) Structural differences were analyzed by calculating the difference in average side chain atom position of 10 highest and 10 lowest ranked models. Blue color indicates low difference, yellow high difference. (B) Sidechain conformations of 10 highest and 10 lowest ranked homology models. Yellow–high ranked models, blue–low ranked models.

Thus, we got interested what determinants could be used to distinguish highly scored models from poorly scored models. First, 10 best and 10 worst performing models were tested for geometric errors like phi-psi outliers and heavy atom clashes in MOE 2015 [24] as well as with homology modeling evaluation programs including VERIFY 3D [25], ERRAT [26] and PROVE [27]. However, none of the applied methods led to a successful discrimination (Fig C in S1 File). Next, we analyzed structural differences by comparing the side chain atoms average position of 10 best and 10 worst performing models (Fig 2). The atom with the highest difference (4.7 Å) in the average position is a carboxyl oxygen of E_5.46_ (Fig 2A). In the highly scored models E_5.46_ is pointing inside the binding pocket (Fig 2B). This is in line with the predominant docking pose that is involved in a hydrogen bond with E_5.46_. In contrast, poorly scored models show a conformation pointing outside the binding pocket. This conformation is also energetically unfavorable, since it is pointing toward the lipophilic membrane and no amino acid with opposite charge is present to compensate the negative charge. The importance of E_5.46_ in ligand binding is in agreement with mutational studies [28] and was already described in previous homology modeling studies for H_3_R [29,30]. Another atom with a rather high difference in mean atom position (2.1 Å) is a distal side chain carbon of L_7.42_. However, we were not able to draw a clear connection to the docking results.

### Virtual screening

**Fig 3:**
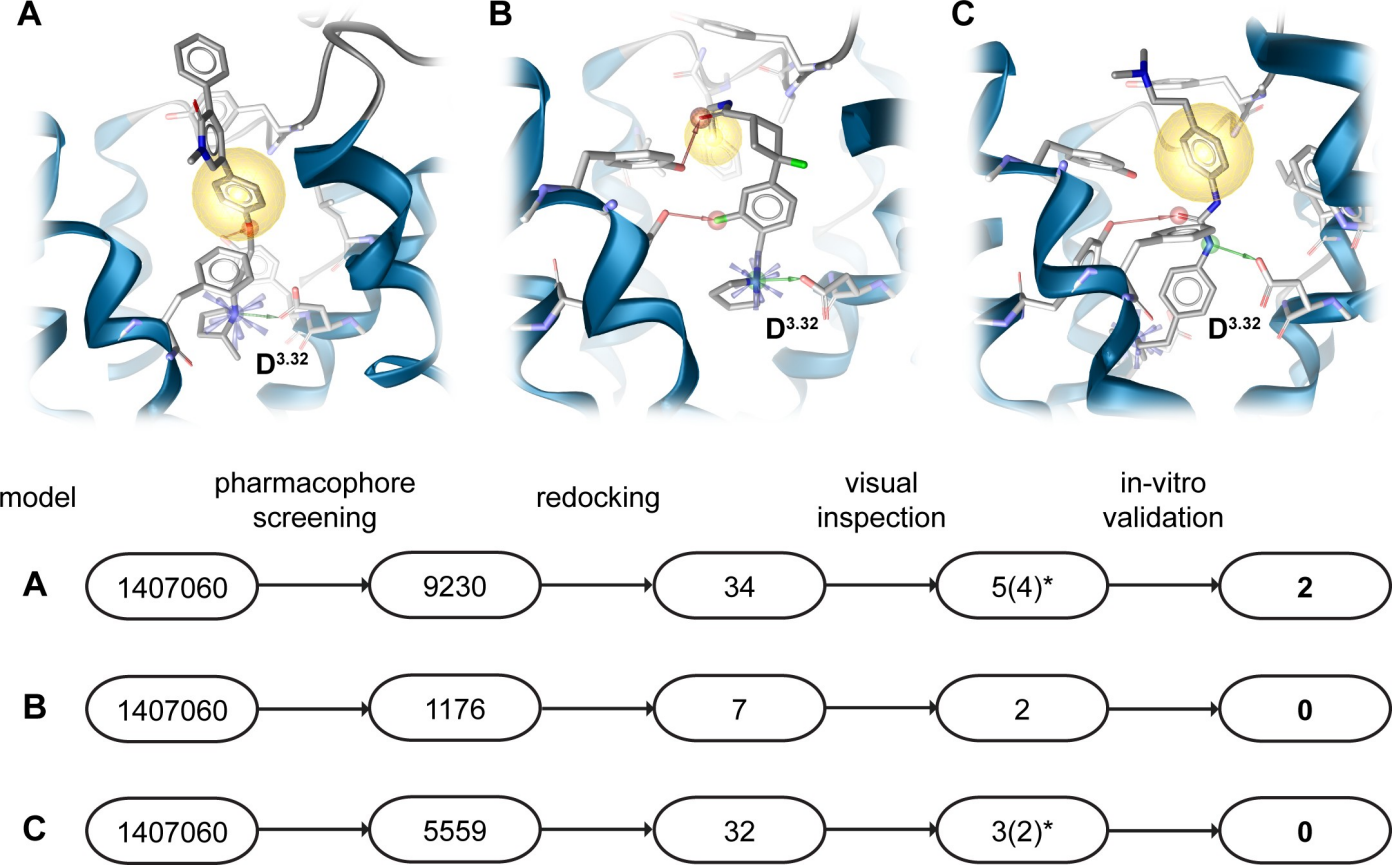
Virtual screening workflow results in 8 compounds out of 1.4 M for in-vitro validation. Workflow for virtual screening using 3 different pharmacophores based on docking poses of CHEMBL1923737 (model A), CHEMBL2151197 (model B) and CHEMBL2387294 (model C). Model A led to identification of compounds 3, 5, 6, 8, 9, model B to compounds 4, 7 and model C to compounds 1, 2, 10 (Fig 4, Table E in S1 File). * compounds 9 and 10 (model A and C) were removed from experimental testing due to insufficient purity as determined by LC-MS. Red arrows–hydrogen bond acceptors, green arrows–hydrogen bond donors, blue star–positive ionizable, yellow sphere–hydrophobic contact.

The highest scored homology model was used for a screening campaign to identify novel H_3_R ligands. 10 diverse antagonists (Table A in S1 File) were docked into the homology model. Constraints were added to focus on docking poses involved in interactions with the negatively charged carboxyl-group of D^3.32^ and E^5.46^, since all inverse agonists contain at least one positively charged group. Docking poses with favorable interaction patterns were found for only 5 out of 10 compounds and additionally analyzed to agree with published structure activity relationship. Derivatives of CHEMBL1923737 (Fig 3, model A) tolerate differently sized pyridone analogues indicating a location of the pyridone group outside the relatively narrow orthosteric binding pocket [31]. The literature about CHEMBL2151197 (Fig 3, model B) has only sparse structure active relationship data [32]. However, later pharmacophore modeling motivated us to include this docking pose in virtual screening. Analogues of CHEMBL2387294 show that 1 positively charged group can be exchanged by hydrophobic groups without loss of activity [33]. Hence, a docking pose was chosen that is extending outside the receptor with more space for different interactions (Fig 3, model C). Data for CHEMBL1269844 report a decrease in activity when attaching the naphthalene moiety in an extending fashion [34]. Concordantly, such molecule would lead to clashes with the receptor in the selected binding mode (Fig D part A in S1 File). The preferred docking pose of the histamine analogue CHEMBL214312 (Fig D part B in S1 File) is complexed between D_3.32_ and E_5.46_ [35]. This binding mode agrees well with several previous docking studies of histamine [29,30]. Each of the 5 chosen binding poses is involved in an interaction with charged residues D_3.32_ and E_5.46_, which is agreement with the common binding mode of aminergic GPCRs involving D_3.32_ and with the importance of E_5.46_ for proper ligand placement in our homology modeling approach (Fig 2) that is further supported by mutational data [28] and previous docking studies [29,30]. Docking poses of CHEMBL1923737, CHEMBL2151197 (Fig 3, model A and B) and CHEMBL1269844 (Fig D part A in S1 File) only interact with D_3.32_ despite the already described importance of E_5.46_ in ligand binding_._ However, CHEMBL1923737 has only a single moiety able to act as hydrogen bond donor. Thus, it can only interact with one of such residues. Additionally, mutational data from the histamine H_1_ receptor suggests that the amino acid at position 5.46 is only important for some ligands [36,37]. Selected complexes were minimized using SZYBKI [38] to allow binding site adaptation to the docked ligand. Pharmacophores were created and iteratively optimized using actives and property-matched decoys generated with DUD-E [39]. Three pharmacophores were found to efficiently discriminate between actives and decoys (Fig 3, Fig E in S1 File). Only pharmacophore model C includes interactions with residue E_5.46_, whose conformation was found to be important for proper ligand docking in prior homology modeling selection. However, the 10 diverse inverse agonists used for this docking differ significantly from the shape of the Y-shaped compounds employed in ligand-guided homology modeling. Thus, it is not surprising that binding modes and interaction partners are to some extent different.

**Fig 4:**
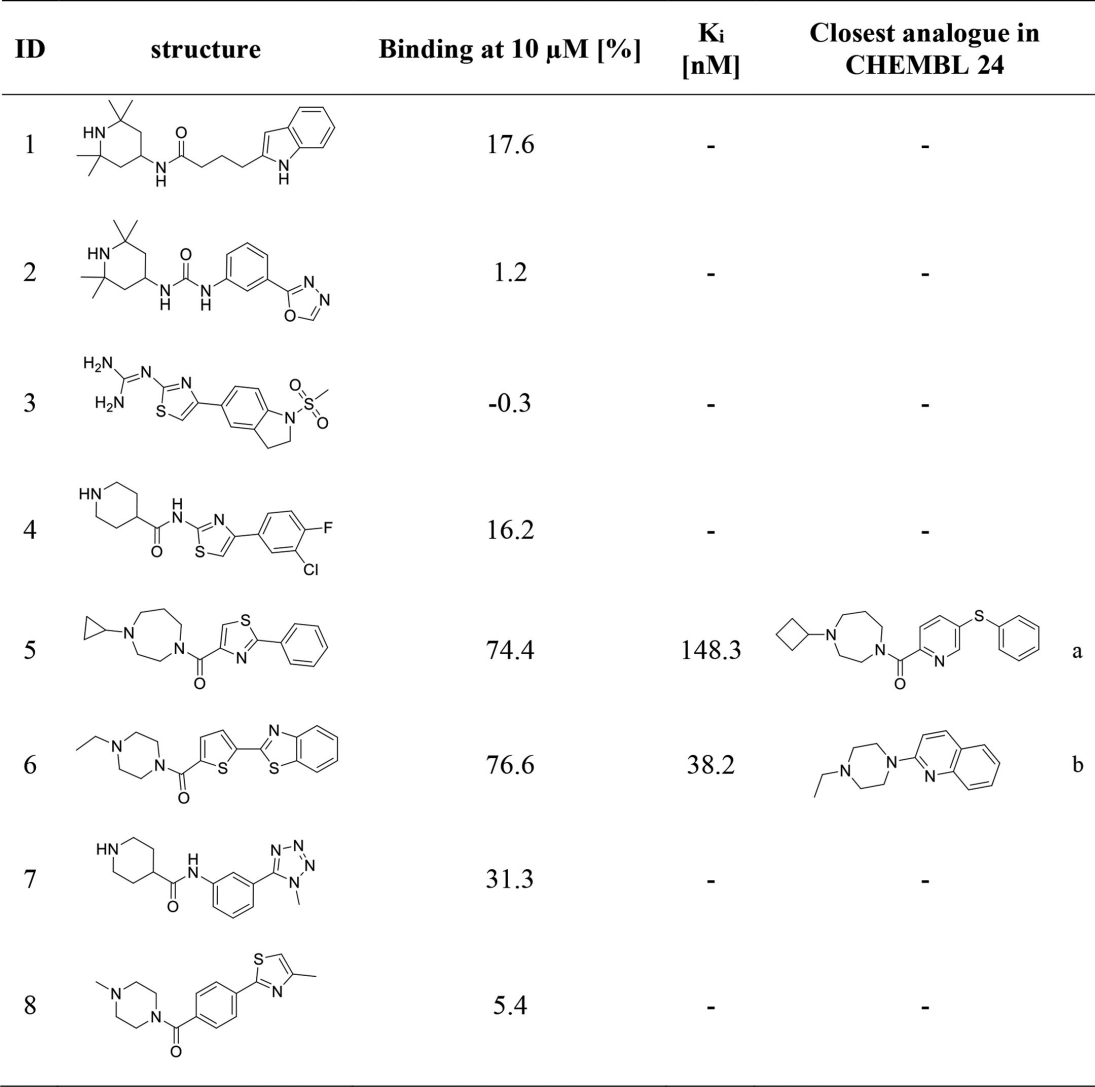
In-vitro validation of virtual screening hits identified 2 novel nanomolar H_3_R ligands. Activity results of radioligand depletion assay against H_3_R. K_i_ data is presented as mean values calculated from at least three independent experiments, each performed in triplicates. ^a^CHEMBL1172076 with Tanimoto score of 0.53 when comparing with compound **5** using Morgan fingerprints, ^b^CHEMBL180478 with Tanimoto score of 0.36 when comparing with compound **6** using Morgan fingerprints.

These pharmacophore models were used to screen a library of 1.4 M commercially available compounds (Enamine Ltd., Kyiv, Ukraine, www.enamine.net) resulting in almost 16,000 hits. The hits were docked into the respective minimized homology model and resulting docking poses were assessed for matching the previously screened pharmacophores. This procedure yielded 73 hits, which were visually inspected to identify hits complementing the receptor binding pocket surface. To broaden the chemical space of H_3_R ligands, hits were also prioritized to cover positive ionizable head groups that are underrepresented or completely absent in the H_3_R ligand data of the CHEMBL 20 database [40], i.e. terminal guanidino, 2,2,6,6-tetramethylpiperidino and secondary amino group (Fig 4). In total, 10 compounds were purchased for in-vitro testing. However, two compounds had to be excluded due to insufficient purity as determined by LC-MS (Table E in S1 File).

**Fig 5:**
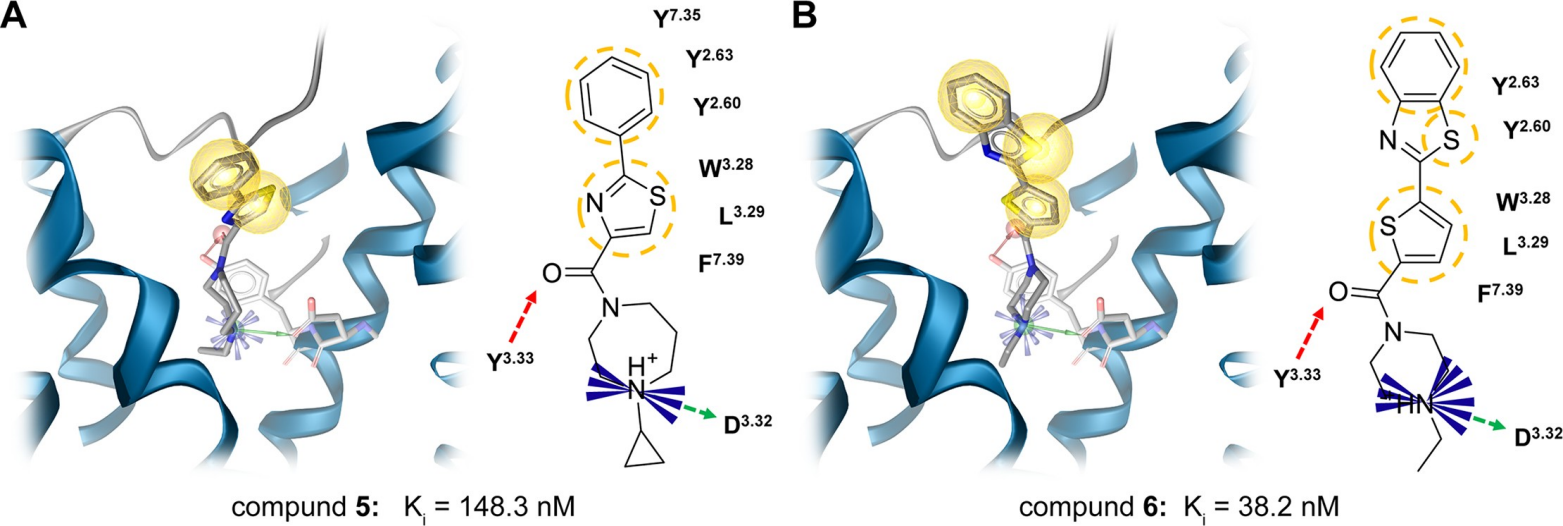
Potential binding modes of active ligands are very similar. Observed interaction of screening hits **5** (A) and **6** (B). Red arrows–hydrogen bond acceptors, green arrows–hydrogen bond donors, blue star–positive ionizable, yellow sphere–hydrophobic contact.

Two molecules (**5** and **6**) were found to bind H_3_R in nanomolar concentration ranges (Fig 5). The identified binding mode indicates very similar interaction patterns including a charged interaction to D_3.32_, hydrogen bonds to D_3.32_ and Y_3.33_ as well as several hydrophobic contacts. Moreover, we observed pi-cation interactions to D_3.32_ and Y_3.33_. Compound **6** shows an additional pi-cation interaction to F_7.39_ which may contribute to its superior activity towards H_3_R compared to compound **5**. Closest H_3_R ligand analogues in CHEMBL 24 [40] were identified by employing Morgan fingerprints [41] implemented in RDKit [42] nodes for KNIME [43] with a Tanimoto score of 0.53 for compound **5** and of 0.36 for compound **6** (Fig 4). The closest analogues were characterized as inverse agonists indicating the same mode of action for the newly identified compounds **5** and **6** [44,45]. According to Morgan fingerprints [41] both compounds significantly differ from CHEMBL1923737 whose docking pose was used for pharmacophore modeling (Table D in S1 File). This is in line with frequently observed scaffold hopping in pharmacophore screening campaigns [46]. The thiazole motif of compound **5** has recently also been incorporated in new lead findings for this receptor subtype [47]. Compound **6** is known as CHEMBL1433079 and was tested in different high throughput bioassays. However, none of the reported primary screen activities was further investigated hindering a proper assessment of the data.

The remaining compounds bound H_3_R at a concentration of 10 μM less than 50% and were not considered for in-depth activity characterization (Fig 4). Compounds **1**–**4** and **7** represent a molecule class that does not carry a lipophilic moiety (e.g. ethyl, cyclopropyl) at the charged head group like in compound **5** and **6** indicating an important role of this structural feature. Compound **8** does carry such hydrophobic moiety at the positively charged amine but was also found to be inactive. Hence, we speculate that the methyl group might be too small to effectively fulfill this structural role.

## Conclusion

In this study, we successfully applied a ligand-guided homology modeling workflow to H_3_R. Therefore, 1000 homology models were generated and evaluated for allowing a charged interaction in ligand docking experiments. A structural analysis of best and worst performing models revealed an important conformation of the binding site residue E_5.46_ that is critical for proper ligand placement by the docking program. The best performing model was subsequently used in a virtual screening campaign and resulted in the identification of 2 novel H_3_R ligands scaffolds with nanomolar affinity. Although successful, we do not claim that the best performing model is necessarily the most realistic one. However, we could show that many models were generated that allowed none or only few docking poses with the characteristic charged interaction. Thus, a single, easy-to-handle descriptor could be used to eliminate many low-quality homology models from further analysis.

## Experimental section

### Preparation of ligand data

The following workflow was conducted in KNIME [43] if not specified else. Histamine H_3_ receptor (H_3_R) ligand data was retrieved from Chembl 20 [40] database and filtered for molecular weight (≤ 500 Da), confidence score (= 9), standard activity type (K_i_, K_d_, IC_50_ or EC_50_), standard relation (=), standard activity value (≤ 10) and standard activity unit (nM). Ligands with unclarified stereo centers were removed with a combination of RDKit [42] and Indigo [48] nodes. If multiple activities were available for a single ligand, binding data (K_i_, K_d_) was preferred over functional data IC_50_ or EC_50_) and more recent data was preferred over older data. The literature of the remaining compounds was checked to remove agonists resulting in a final set of 632 inverse agonists. From this set 10 diverse inverse agonists (Table A in S1 File) were selected using the RDKit diversity picker based on MorganFeat fingerprints [41] (diameter = 4). This set was used for docking experiments to generate pharmacophores. Additionally, 100 diverse inverse agonists were selected for pharmacophore validation. Furthermore, the 100 diverse inverse agonists were used to generate decoys using the DUD-E decoy generator [39] for pharmacophore validation. The decoy set contains 3051 unique molecules. 3D coordinates of all molecules used in this study were generated and energetically minimized with the MMFF94s [49] force field using RDKit nodes[42]. Hydrogens were added, strong acids deprotonated and strong bases protonated by using the molecule wash function in MOE 2015 [24].

### Homology modeling

The amino acid sequence of human H_3_R was retrieved from Uniprot [50] (Q9Y5N1) and employed for a homology model template search in the PDB[51] using the BLAST algorithm [52]. Structure files of the top ranked templates in the inactive conformation were used for an alignment in MOE 2015 [24]. Surprisingly, the crystal structure of H_1_R (3RZE [16]) did not show the highest sequence similarity to H_3_R. Also, the extracellular loop 2 (ECL2) close to the orthosteric binding pocket is not resolved in the H_1_R structure. Hence, homology modeling was performed with a multiple-template approach. MODELLER 9.15 [17] was used to generate 1000 homology models using H_1_R (3RZE [16]), muscarinic M_2_ receptor (M_2_R, 3UON [53]) and muscarinic M_3_ receptor (M_3_R, 4U15 [54]) as templates. Since ECL2 is not completely resolved in the H_1_R structure (3RZE), unresolved ECL2 parts were built by MODELLER solely based on M_2_R (3UON) and M_3_R (4U15). The sequence alignment as well as changed parameters of MODELLER functions can be found in the supporting information (Fig A and Table B in S1 File).

### Docking experiments

A set of 9 inverse agonists [19] (Table C in S1 File) was chosen to guide the homology model selection and docked into all homology models using GOLD 5.2 [55] with default settings if not specified otherwise. The active site was defined by residues that are known from other aminergic GPCRs to be involved in ligand binding (D_3.32_, Y_3.33_, Y_4.56_, E_5.46_, W_6.48_, Y_6.51_ and P_7.39_) [11]. 10 conformations were generated per molecule with the genetic algorithm set to 'Library Screening'. Early termination was disabled resulting in 90 conformations per homology model. Docking results were analyzed for ionic interaction between the ligand and D_3.32_ that is characteristic for aminergic GPCRs [11]. Less or equal than 6 Å between the carbon atom of the carboxyl group of D_3.32_ and the positively charged amine of the ligand was considered to be sufficient for ionic interaction. Docking and scoring have been performed to twice to control for variation introduced by the docking algorithm (Fig B in S1 File).

### Homology model evaluation

10 best and 10 worst performing models were tested for geometric errors like phi-psi outliers and heavy atom clashes in MOE 2015 [24] as well as with homology modeling evaluation programs including VERIFY 3D [25], ERRAT [26] and PROVE [27]. No statistically significant difference was found (Fig C in S1 File).

### Pharmacophore generation

The 10 diverse H_3_R inverse agonists generated as described above were docked into the selected homology model using GOLD 5.2 [55] with default settings if not specified otherwise. The active site was defined by residues that are known from other aminergic GPCRs to be involved in ligand binding(D_3.32_, Y_3.33_, Y_4.56_, E_5.46_, W_6.48_, Y_6.51_ and P_7.39_) [11]. 10 conformations were generated per molecules with flip ring corners, flip pyramidal N and generate diverse solutions settings enabled and early termination setting disabled. Protein HBond constraints with a constraint weight of 10 and a minimum H-bond geometry weight of 0.005 were added to focus on conformations involving hydrogen bonds to carboxyl oxygens of D^3.32^ and E^5.46^, since all docked ligands contain a positively charged group that should interact with negatively charged carboxyl-group of D^3.32^ or E^5.42^. Docking results were analyzed in LigandScout 3.12 [56] for interactions explaining the structure-activity relationship. Selected complexes were minimized using Szybki 1.8.0.1 [38] with the MMFF94s forcefield and the Poisson-Boltzmann model. Sidechains within 10 Å were set flexible to allow adaption of the binding site residues to the docked ligand. LigandScout 3.12 was used to generate pharmacophores of the minimized complexes. Default pharmacophores generated with LigandScout 3.12 were optimized against a set of 100 diverse active inverse agonists and 3051 decoys by removing features or increasing the tolerance radius of selected features if supported by the structure activity relationship. Three pharmacophores were found to successfully discriminate between actives and decoys according to receiver operating characteristic curves (Fig E in S1 File).

### Virtual screening and selection

The three selected pharmacophores were employed to screen a library of 1464080 molecules (Enamine Ltd., Kyiv, Ukraine, www.enamine.net) using LigandScout 3.12 [56] resulting in 15965 hits. These hits were redocked into the respective minimized model using GOLD 5.2 with default settings if not specified otherwise. The active site was defined by residues that are known from other aminergic GPCRs to be involved in ligand binding (D_3.32_, Y_3.33_, Y_4.56_, E_5.46_, W_6.48_, Y_6.51_ and P_7.39_) [11]. 10 conformations were generated per molecules with flip ring corners, flip pyramidal N and generate diverse solutions settings enabled. The redocked poses were scored to match the features of the respective pharmacophore model resulting in 73 hits. This set was visually inspected, and 10 molecules were selected for purchase. Ordered compounds were analyzed for purity with LC-MS leading to exclusion of 2 molecules from further analysis. The 8 remaining molecules possess purities of at least 95% and were tested in-vitro for activity against H_3_R (Table E in S1 File).

### In-vitro experiments

Radioligand depletion assays were performed as described previously using crude hH_3_R membrane extracts obtained from HEK-293 cells stably expressing the hH_3_R [15,57]. Briefly, crude membrane extracts were incubated with various concentrations of test ligands (between 0.01 nM and 100 μM) and [3H]-N-alpha-methylhistamine. Bound radioligand were harvested through GF/B filters and measured using liquid scintillation counting. Data analysis were performed with GraphPad Prism 6 using non-linear regression. The Ki values for each experiment were obtained according to Cheng-Prusoff and converted to pKi values to allow statistical analysis. Mean values were calculated from at least three independent experiments, each performed in triplicates (Table E in S1 File).

## Supporting information

S1 File PDF File with used molecular structures as well as more detailed parameters and results. (PDF)
