# Supplementary material for: Ligand-guided homology modeling drives identification of novel histamine H3 receptor ligands
Source: PLoS One. 2019 Jun 25;14(6):e0218820. doi: 10.1371/journal.pone.0218820 (PMC6592549; doi:10.1371/journal.pone.0218820)
Supplement: S1 File — (PDF) [file pone.0218820.s001.pdf]

Supporting Information

Ligand-guided homology modeling drives identification of novel ligands of histamine H3 receptor

David Schaller, Stefanie Hagenow, Holger Stark and Gerhard Wolber

Table of contents

1. Computational experiments 1

1.1 Preparation of ligand data 1

1.2 Homology modeling 2

1.3 Docking experiments 4

1.4 Homology model evaluation 5

1.5 Additional binding modes not used for pharmacophore screening 6

1.6 ROC plot analysis of pharmacophores used for virtual screening 7

1.6 Similarity matrix 8

2. In-vitro experiments 9

3. References 10

1. Computational experiments

1.1 Preparation of ligand data

Table A: Diverse H_3R ligands. 10 diverse ligands used for docking experiments in selected homology model.

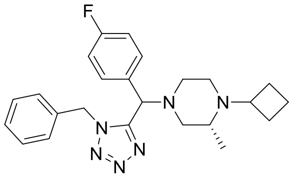 CHEMBL1222946, IC_{50}=2.2 nM[1]	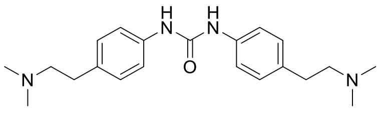 CHEMBL2387294, IC_{50}=6.5 nM[2]	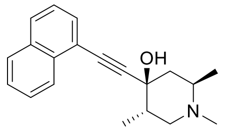 CHEMBL1269844, IC_{50}=9 nM[3]
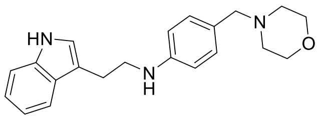 CHEMBL2419581, K_i=4.3 nM[4]	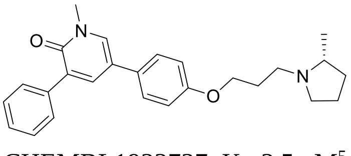 CHEMBL1923737, K_i=3.5 nM[5]	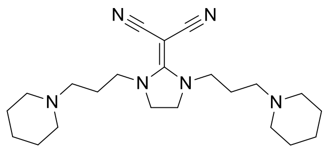 CHEMBL257391, K_i=2.4 nM[6]
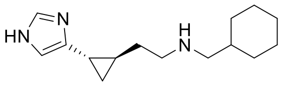 CHEMBL214312, K_i=5.3 nM[7]	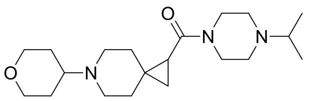 CHEMBL3124968, IC_{50}=8.3 nM[8]	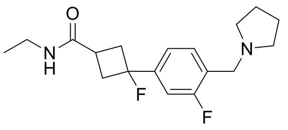 CHEMBL2151197, K_i=2.3 nM[9]
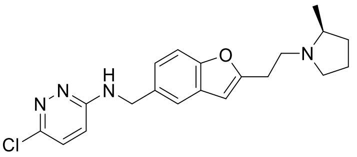 CHEMBL362952, K_i=0.3 nM[10]		

1.2 Homology modeling

```
                   <-----------H1----------->      <-------------H2----------
H3R          35   AVLAALMALLIVATVLGNALVMLAFVADSSLRTQNNFFLLNLAISDFLVGAFCIPLYVPY  94
H1R (3RZE)        MPLVVVLSTICLVTVGLNLLVLYAVRSERKLHTVGNLYIVSLSVADLIVGAVVMPMNILY
M2R (3UON)        VFIVLVAGSLSLVTIIGNILVMVSIKVNRHLQTVNNYFLFSLACADLIIGVFSMNLYTLY
M3R (4U15)        VFIAFLTGFLALVTIIGNILVIVAFKVNKQLKTVNNYFLLSLACADLIIGVISMNLFTTY
                    :. : . : :.*:  * **: :.  :  *:*  * ::..*: :*:::*.. : :   *
                                            <ICL1>

                  ->      <----------------H3--------------->          <-----
H3R          95   VLTGRWTFGRGLCKLWLVVDYLLCTSSAFNIVLISYDRFLSVTRAVSYRAQQGDTRRAVR  154
H1R (3RZE)        LLMSKWSLGRPLCLFWLSMDYVASTASIFSVFILCIDRYRSVQQPLRYL-KYRTKTRASA
M2R (3UON)        TVIGYWPLGPVVCDLWLALDYVVSNASVMNLLIISFDRYFCVTKPLTYP-VKRTTKMAGM
M3R (4U15)        IIMNRWALGNLACDLWLSIDYVASNASVMNLLVISFDRYFSITRPLTYR-AKRTTKRAGV
                    :   * :*   * :** :**: ..:* :.:.::. **: .: : : *    ...  *
                    <ECL1>                                 <--ICL2--->

                  ------H4--------------->               <-------------H5---
H3R         155   KMLLVWVLAFLLYGPAILSWEYLSGGSSIPEGHCYAEFFYNWYFLITASTLEFFTPFLSV  214
H1R (3RZE)        TILGAWFLSF-LWVIPILGWNH/----/RREDKCETDFYDVTWFKVMTAIINFYLPTLLM
M2R (3UON)        MIAAAWVLSFILWAPAILFWQFIVGVRTVEDGECYIQFFSNAAVTFGTAIAAFYLPVIIM
M3R (4U15)        MIGLAWVISFVLWAPAILFWQYFVGKRTVPPGECFIQFLSEPTITFGTAIAAFYMPVTIM
                    :  .*.::* *:    ** *:.          .*  :*     . . ::    *: *   :
                                            <-----ECL2------>

                  ---------->    <--------------H6--------------->       <----
H3R         215   TFFNLSIYLNIQR/LSRDRKVAKSLAVIVSIFGLCWAPYTLLMIIRAACHGHCVPDYWYE  395
H1R (3RZE)        LWFYAKIYKAVRQ/MNRERKAAKQLGFIMAAFILCWIPYFIFFMVIAFCKN-CCNEHLHM
M2R (3UON)        TVLYWHISRASKS/PSREKKVTRTILAILLAFIITWAPYNVMVLINTFCAP-CIPNTVWT
M3R (4U15)        TILYWRIYKETEK/LIKEKKAAQTLSAILLAFIITWTPYNIMVLVNTFCDS-CIPKTYWN
                     :   *     .    :::*.:: :   *:   * : * ** ::.:: : *   * .
                            <ICL3>                                  <-ECL3->

                  -------H7----------> <---H8--->
H3R         396   TSFWLLWANSAVNPVLYPLCHHSFRRAFTKL  426
H1R (3RZE)        FTIWLGYINSTLNPLIYPLCNENFKKTFKRI
M2R (3UON)        IGYWLCYINSTINPACYALCNATFKKTFKHL
M3R (4U15)        LGYWLCYINSTVNPVCYALCNKTFRTTFKTL
                     ** : **::**  * **. .*: :*. :
```

Fig A: Sequence alignment. The depicted multiple sequence alignment was used for ligand-guided homology modeling of human H$_3$R. The template sequences are illustrated according to structural properties. Red sections represent helices and underlined cysteines are involved in a disulfide bond. Furthermore, the multiple sequence alignment contains information about the naming of helices and loops and the sequence similarity. (H1) - helix 1, (ILC1) – intracellular loop 1, (ECL1) – extracellular loop 2, (*) - identical residues, (:) - residues with high similarity, (.) - residues with low similarity.

Table B: MODELLER parameters. Changed parameters of environ and allhmodel classes in homology modelling using MODELLER 9.15[11].

class	parameter	setting
environ	schedule_scale	physical.values(default=1.0, soft_sphere=0.7)
allhmodel	library_schedule	autosched.slow
allhmodel	max_var_iterations	300
allhmodel	md_level	refine.slow
allhmodel	repeat_optimization	2
allhmodel	max_molpdf	1e6

1.3 Docking experiments

Table C: Homology modeling ligand series. Structures of molecules used for guiding homology model selection. Provided activities are taken from the literature[12].

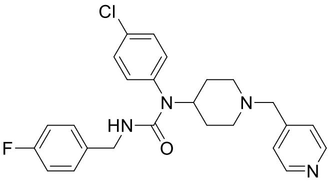 K_i=16 nM	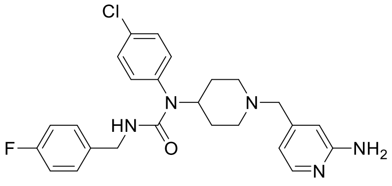 K_i=3 nM	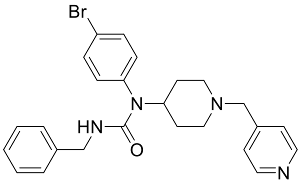 K_i=2 nM
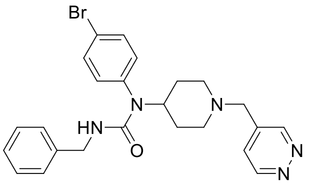 K_i=10 nM	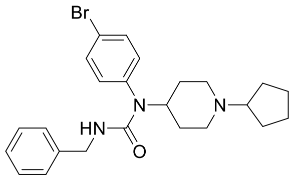 K_i=10 nM	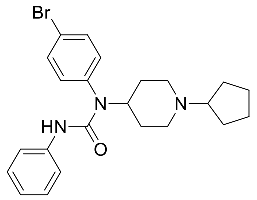 K_i=14 nM
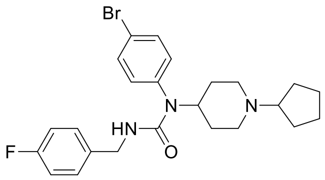 K_i=7 nM	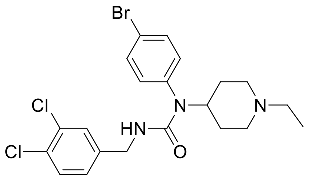 K_i=23 nM	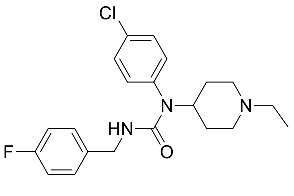 K_i=7 nM

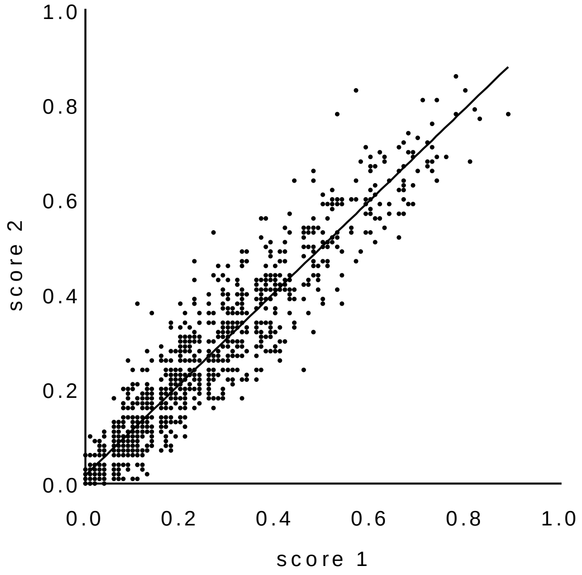

Fig B: Scoring repeats. Scoring results from two independent docking experiments with linear regression line (R^2 = 0.89).

1.4 Homology model evaluation

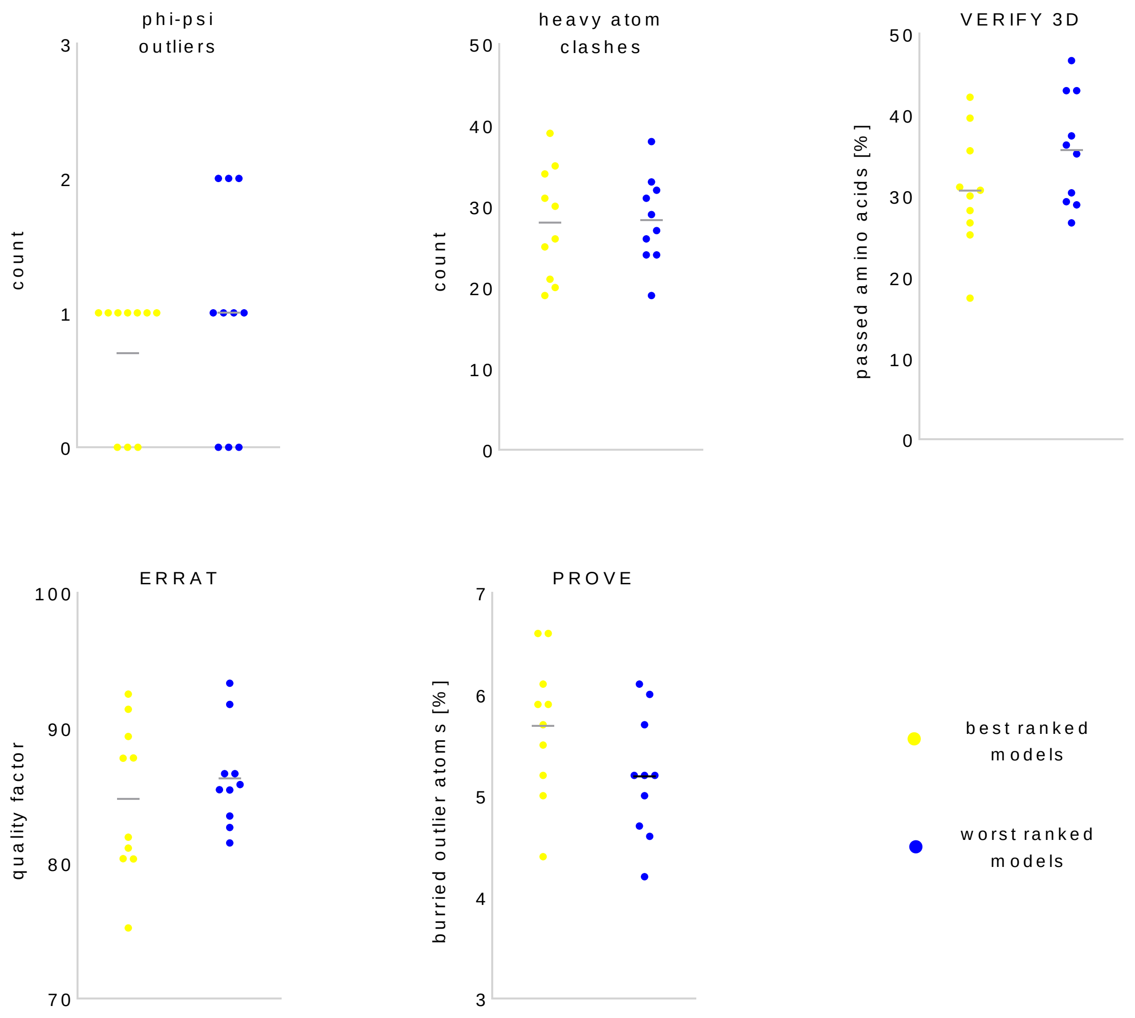

Fig C: Homology model evaluation. Model quality comparison of 10 best and 10 worst performing models using Mann-Whitney U test as implemented in GraphPad Prism 6.

1.5 Additional binding modes not used for pharmacophore screening

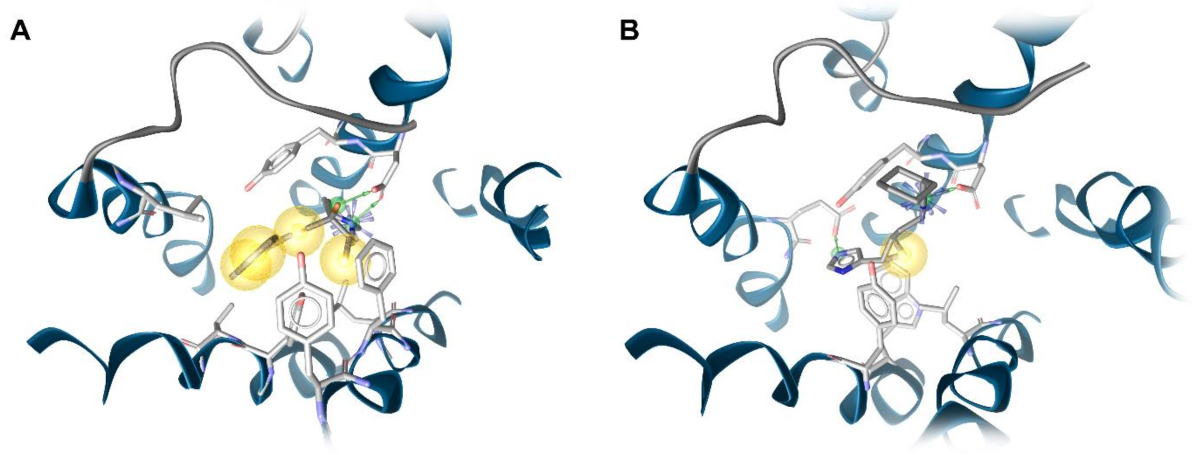

Fig D: Additional proposed binding modes of docked diverse antagonists for CHEMBL1269844[3] and CHEMBL214312[7]. Green arrows – hydrogen bond donors, blue star – positive ionizable, yellow sphere – hydrophobic contact.

1.6 ROC plot analysis of pharmacophores used for virtual screening

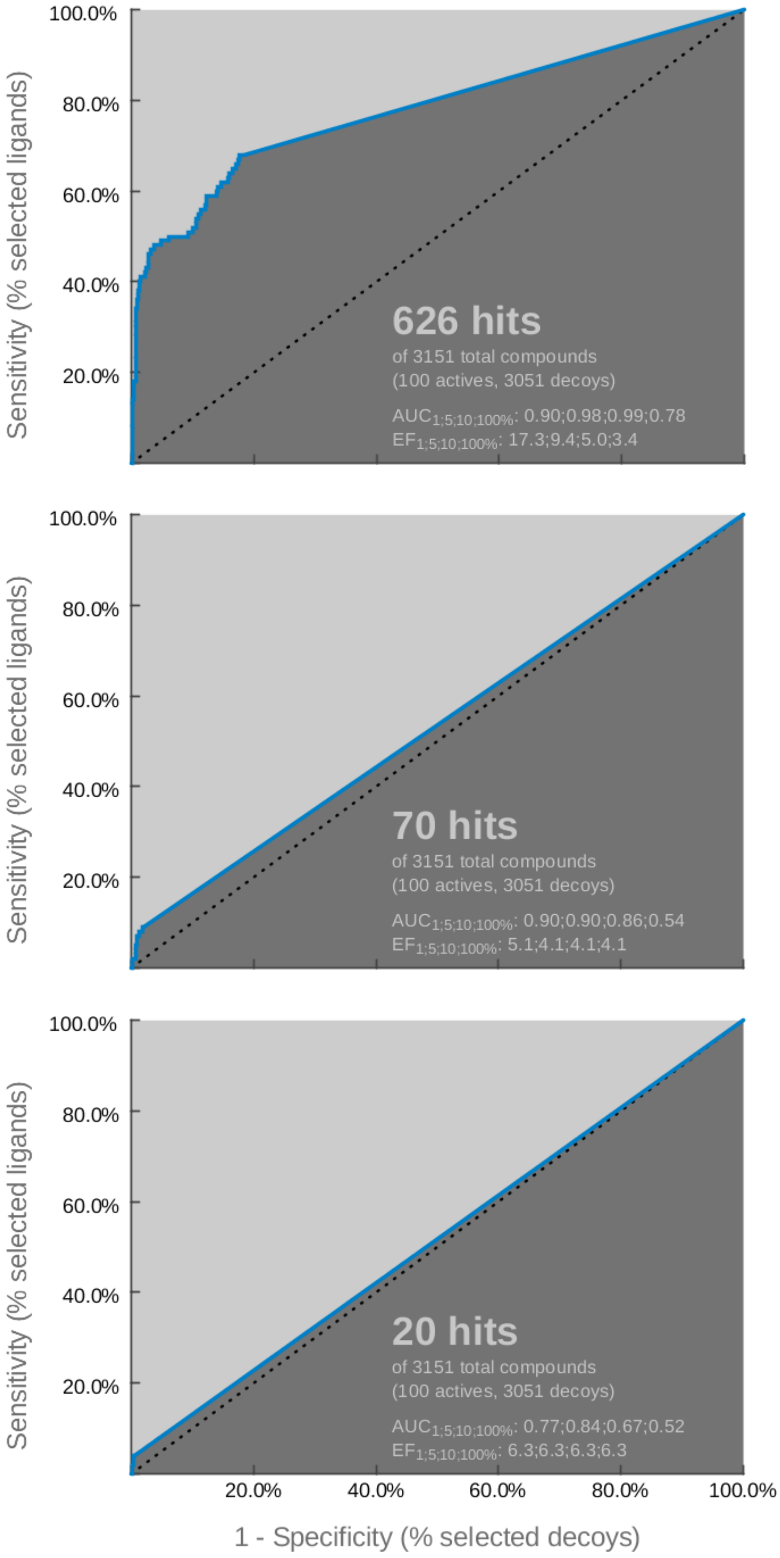

Fig E: ROC plot analysis of pharmacophores for virtual screening. (Top) Model A, CHEMBL1923737, (Middle) Model B, CHEMBL2151197, (Bottom) CHEMBL2387294.

1.6 Similarity matrix

Table D: Similarity matrix between 10 diverse already known H_3R ligands and 2 newly identified H_3R ligands. Similarities are represented as Tanimoto scores calculated with Morgan fingerprints[13] as implanted in RDKit[14] nodes in KNIME[15].

	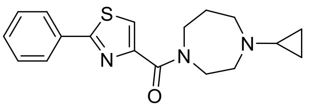	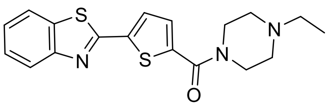
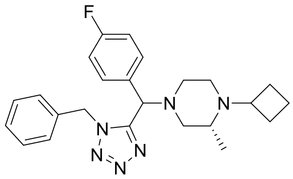	0.190	0.161
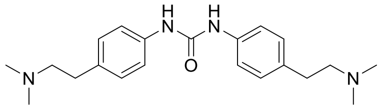	0.109	0.125
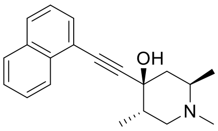	0.149	0.147
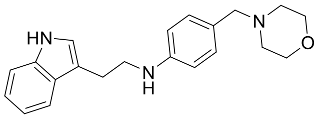	0.127	0.139
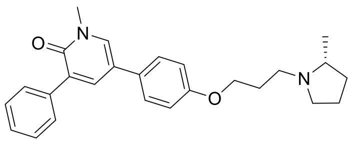	0.188	0.129
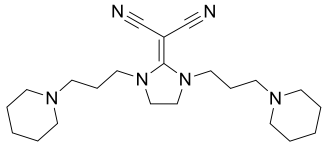	0.09	0.121
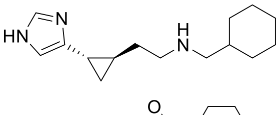	0.139	0.107
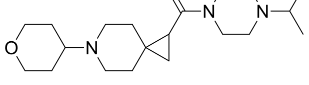	0.200	0.118
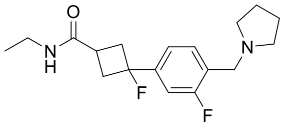	0.139	0.167
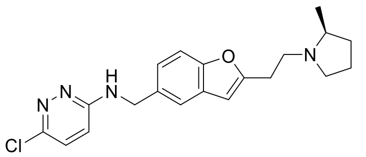	0.151	0.136

2. In-vitro experiments

Table E: In-vitro results and purity. Purity and affinity of tested compounds analyzed with LC-MS and radioligand depletion experiment, respectively.

**ID**	**structure**	**purity [%]**	**MW [g/mol]**	**m/z $[M+H^+]$**	**Binding at 10 µM [%]**	**pK_i $\bar{x}$ ± SEM**
1	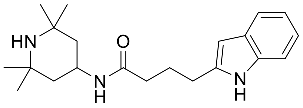	> 95	341.2	342.3	17.6	-
2	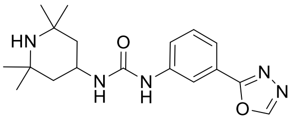	> 95	343.2	344.3	1.2	-
3	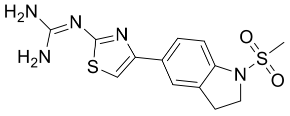	> 95	337.1	338.0	-0.3	-
4	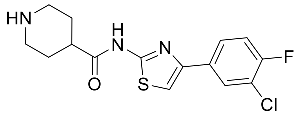	> 95	339.1	340.0	16.2	-
5	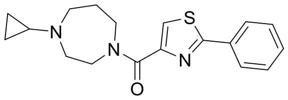	> 95	327.1	328.0	74.4	**6.83 ± 0.23**
6	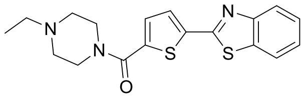	> 95	357.1	358.0	76.6	**7.46 ± 0.11**
7	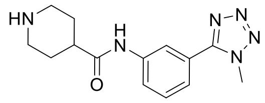	> 95	286.2	287.3	31.3	-
8	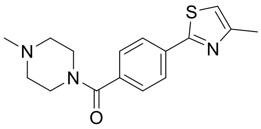	> 95	301.1	302.0	5.4	-
9	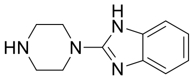	**< 90**	202.1	203.0	[a]	
10	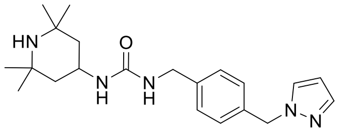	**< 90**	369.3	370.3	[a]	

[a] Excluded due to insufficient purity.

3. References

(1) Davenport, A. J.; Stimson, C. C.; Corsi, M.; Vaidya, D.; Glenn, E.; Jones, T. D.; Bailey, S.; Gemkow, M. J.; Fritz, U.; Hallett, D. J. Discovery of Substituted Benzyl Tetrazoles as Histamine H3 Receptor Antagonists. *Bioorg. Med. Chem. Lett.* **2010**, *20* (17), 5165–5169.

(2) Gao, Z.; Hurst, W. J.; Guillot, E.; Czechtizky, W.; Lukasczyk, U.; Nagorny, R.; Pruniaux, M. P.; Schwink, L.; Sanchez, J. A.; Stengelin, S.; Tang, L.; Winkler, I.; Hendrix, J. a.; George, P. G. Discovery of Aryl Ureas and Aryl Amides as Potent and Selective Histamine H3 Receptor Antagonists for the Treatment of Obesity (Part I). *Bioorganic Med. Chem. Lett.* **2013**, *23* (11), 3416–3420.

(3) Anderson, J. T.; Campbell, M.; Wang, J.; Brunden, K. R.; Harrington, J. J.; Stricker-Krongrad, A.; Song, J.; Doucette, C.; Murphy, S.; Bennani, Y. L. Investigation of 4-Piperidinols as Novel H3 Antagonists. *Bioorg. Med. Chem. Lett.* **2010**, *20* (21), 6246–6249.

(4) Tang, L.; Zhao, L.; Hong, L.; Yang, F.; Sheng, R.; Chen, J.; Shi, Y.; Zhou, N.; Hu, Y. Design and Synthesis of Novel 3-Substituted-Indole Derivatives as Selective H3 Receptor Antagonists and Potent Free Radical Scavengers. *Bioorg. Med. Chem.* **2013**, *21* (19), 5936–5944.

(5) Becknell, N. C.; Lyons, J. A.; Aimone, L. D.; Gruner, J. A.; Mathiasen, J. R.; Raddatz, R.; Hudkins, R. L. Synthesis and Evaluation of Pyridone-Phenoxypropyl-R-2-Methylpyrrolidine Analogues as Histamine H3 Receptor Antagonists. *Bioorg. Med. Chem. Lett.* **2011**, *21* (23), 7076–7080.

(6) Sasho, S.; Seishi, T.; Kawamura, M.; Hirose, R.; Toki, S.; Shimada, J. Diamine Derivatives Containing Imidazolidinylidene Propanedinitrile as a New Class of Histamine H3 Receptor Antagonists. Part I. *Bioorg. Med. Chem. Lett.* **2008**, *18* (7), 2288–2291.

(7) Watanabe, M.; Kazuta, Y.; Hayashi, H.; Yamada, S.; Matsuda, A.; Shuto, S. Stereochemical Diversity-Oriented Conformational Restriction Strategy. Development of Potent Histamine H3 and/or H4 Receptor Antagonists with an Imidazolylcyclopropane Structure. *J. Med. Chem.* **2006**, *49* (18), 5587–5596.

(8) Brown, D. G.; Bernstein, P. R.; Griffin, A.; Wesolowski, S.; Labrecque, D.; Tremblay, M. C.; Sylvester, M.; Mauger, R.; Edwards, P. D.; Throner, S. R.; Folmer, J. J.; Cacciola, J.; Scott, C.; Lazor, L. A.; Pourashraf, M.; Santhakumar, V.; Potts, W. M.; Sydserff, S.; Giguère, P.; Lévesque, C.; Dasser, M.; Groblewski, T. Discovery of Spirofused Piperazine and Diazepane Amides as Selective Histamine-3 Antagonists with in Vivo Efficacy in a Mouse Model of Cognition. *J. Med. Chem.* **2014**, *57* (3), 733–758.

(9) Wager, T. T.; Pettersen, B. A.; Schmidt, A. W.; Spracklin, D. K.; Mente, S.; Butler, T. W.;

Howard, H.; Lettiere, D. J.; Rubitski, D. M.; Wong, D. F.; Nedza, F. M.; Nelson, F. R.; Rollema, H.; Raggon, J. W.; Aubrecht, J.; Freeman, J. K.; Marcek, J. M.; Cianfrogna, J.; Cook, K. W.; James, L. C.; Chatman, L. A.; Iredale, P. A.; Banker, M. J.; Homiski, M. L.; Munzner, J. B.; Chandrasekaran, R. Y. Discovery of Two Clinical Histamine H(3) Receptor Antagonists: Trans-N-Ethyl-3-Fluoro-3-[3-Fluoro-4-(Pyrrolidinylmethyl)Phenyl]Cyclobutanecarboxamide (PF-03654746) and Trans-3-Fluoro-3-[3-Fluoro-4-(Pyrrolidin-1-Ylmethyl)Phenyl]-N-(2-Methylpropyl)Cyclobuta. *J. Med. Chem.* **2011**, *54* (21), 7602–7620.

(10) Sun, M.; Zhao, C.; Gfesser, G. A.; Thiffault, C.; Miller, T. R.; Marsh, K.; Wetter, J.; Curtis, M.; Faghih, R.; Esbenshade, T. A.; Hancock, A. A.; Cowart, M. Synthesis and SAR of 5-Amino- and 5-(Aminomethyl)Benzofuran Histamine H3 Receptor Antagonists with Improved Potency. *J. Med. Chem.* **2005**, *48* (20), 6482–6490.

(11) Webb, B.; Sali, A. Comparative Protein Structure Modeling Using MODELLER. *Curr. Protoc. Bioinforma.* **2016**, *54*, 5.6.1-5.6.37.

(12) Berlin, M.; Lee, Y. J.; Boyce, C. W.; Wang, Y.; Aslanian, R.; McCormick, K. D.; Sorota, S.; Williams, S. M.; West, R. E.; Korfmacher, W. Reduction of HERG Inhibitory Activity in the 4-Piperidinyl Urea Series of H3 Antagonists. *Bioorg. Med. Chem. Lett.* **2010**, *20* (7), 2359–2364.

(13) Rogers, D.; Hahn, M. Extended-Connectivity Fingerprints. *J. Chem. Inf. Model.* **2010**, *50* (5), 742–754.

(14) RDKit: Open-Source Cheminformatics; Http://Www.Rdkit.Org.

(15) Berthold, M. R.; Cebron, N.; Dill, F.; Gabriel, T. R.; Kötter, T.; Meinl, T.; Ohl, P.; Sieb, C.; Thiel, K.; Wiswedel, B. KNIME: The Konstanz Information Miner; 2008; pp 319–326.
